# Queer Aging: Older Lesbian, Gay, and Bisexual Adults’ Visions of Late Life

**DOI:** 10.1093/geroni/igad021

**Published:** 2023-03-02

**Authors:** Lisa R Miller

**Affiliations:** Department of Sociology, Behavioral Sciences, Eckerd College, Saint Petersburg, Florida, USA

**Keywords:** Aging in community, Aging in place, Death and dying, End-of-life care, LGBTQ aging

## Abstract

**Background and Objectives:**

Diversity in aging has received increased attention in recent years in the field of gerontology. However, older lesbian, gay, and bisexual (LGB) people have largely been missing from these conversations. In this study, I examine older LGB people’s subjective views on the aging process, focusing specifically on visions of late life.

**Research Design and Methods:**

Life story interviews were conducted with 60 LGB individuals over the age of 55 who reside in the Southeastern and Midwestern portions of the United States. Inductive coding (e.g., line-by-line, focused) and analyses were conducted.

**Results:**

Four major themes emerged from the data: (1) financial distress linked to past events of homophobic discrimination, (2) anxieties regarding staying in paid care settings, (3) desires to age in place or “in community” with other lesbian, gay, bisexual, transgender, and queer people, and (4) a prioritization of quality of life over longevity via plans to pursue assisted suicide.

**Discussion and Implications:**

The findings of this study suggest that views of aging and visions of late life are tied to social group membership, highlighting the need for gerontologists to further consider cumulative inequality processes. The study also offers evidence of queer aging, wherein queer culture, history, and experience produce distinct meanings of aging.

**Translational Significance:** Heightened aging-related challenges were reported in a qualitative sample of older lesbian, gay, and bisexual (LGB) individuals. Understanding older LGB people’s fears about aging and the utilization of paid care services is necessary in order to improve the quality of care that this population receives in late adulthood. In particular, staff in health care settings and long-term care homes should be educated on how to provide culturally competent care, including the usage of inclusive terminology.

## Background and Objectives

There are at least 2.7 million lesbian, gay, and bisexual (LGB) adults over the age of 50 in the United States, and this number is expected to reach nearly 5 million in the next few decades (Fredriksen-Goldsen, 2016). Yet, older individuals remain an invisible segment of the LGB community and within lesbian, gay, bisexual, transgender, and queer (LGBTQ) studies more broadly (Lytle et al., 2018; Torres & Lacy, 2021). LGB people are simultaneously overlooked in discussions of older adults, including in the fields of aging and gerontology. Additionally, a great deal of social change in the area of gay rights has occurred during the lifetime of older LGB people, presenting both opportunities and challenges for their lives (Thomeer et al., 2017). Consequently, the lives of older LGB people warrant much greater scholarly attention.

### The Process of Aging

I begin by describing issues that generally accompany the aging process. Poverty is not uncommon in late adulthood, due to a loss of income as a consequence of retirement; similarly, individuals may not have adequate savings for their retirement years (Victor, 2005). In addition, aging entails physiological and biological processes that may result in functional limitations, the onset of illness and disease, and declines in cognitive functioning (Cicero & Pynoos, 2016; Mogle & Sliwinski, 2013). It is also not uncommon for older adults’ social networks to shrink, especially as their peers and family begin to pass; consequently, older adults sometimes experience loneliness and depression (Fingerman et al., 2013). Simultaneously, there has been a recent trend in the field to consider successful, positive, and resilient aspects of aging (Miller, 2019; Minichiello & Clulson, 2005; Rowe & Kahn, 1998).

Scholars in recent years also investigate diversity in aging, emphasizing that gender, race, and social class shape aging experiences (Arber et al., 2003; Calasanti & Slevin, 2001; Mehrotra & Wagoner, 2019; Settersten & Trauten, 2009). Central to understanding variability in aging experiences is the cumulative inequality approach, which posits that the accumulation of inequality over the life course leads to worsened quality of life in late adulthood (Dannefer, 2003; Ferraro & Shippee, 2009). This theory also asserts that early life events shape outcomes in late adulthood, including health (Ferraro & Shippee, 2009; White et al., 2020). Far less, however, is known about how sexual identity-based inequalities shape the aging process (Barbee, 2022; Lytle et al., 2018; Minkler & Estes, 2020).

### Sexual Identity Differences in Aging and the Life Course

Relying on the traditions of critical gerontology and queer theory, I develop the concept of *queer aging* to suggest that the meaning of aging among LGB adults may be distinct (Fabbre, 2014; Fabbre et al., 2019). The term “queer” is used in this article to signify a nonnormative way of being, consistent with its usage within queer theory. Queer theory questions and destabilizes normative and traditional ideas about gender and sexual expressions and identities (Butler, 1990; Fabbre, 2014; Halberstam, 2005; Sedgwick, 1990). Queer theorists also emphasize the distinctiveness of queer culture and lives, which in part developed out of systematic exclusion from the institution of marriage and family (Fabbre, 2014; Fredriksen, 2016). For example, due to estrangement from biological family, LGB people formed their own unique subculture, rejecting biological and reproductive definitions of kinship; in turn, they embraced friends as “chosen family” and as a source of support (de Vries et al., 2019; Weston, 1997). Despite pressures to assimilate into mainstream culture given the liberalization of same-sex marriage (Duggan, 2002; Hull & Ortyl, 2019; Warner, 1999), some members of the LGB community retain distinctive features of queer subculture, including continuing to embrace “chosen family” (Hull & Ortyl, 2019). Thus, queer subculture has implications for how LGB people view the future (Edelman, 2004; Sandberg & Marshall, 2017), including visions for late life.

*Queer aging* also entails the recognition that queer subculture may shift how temporarily and time are experienced (Edelman, 2004; Fabbre, 2014; Halberstam, 2005). For example, LGB people often violate societal expectations about when major life events should occur; these normative sequencing expectations are known as chronormativity (Freeman, 2010). Indeed, LGB people have not historically met heterosexual-centric markers of adulthood (i.e., marriage, childrearing) and, in other cases, these major life events were delayed until fairly recently (Fredriksen-Goldsen & Kim, 2017; Freeman, 2010; Halberstam, 2005; Rosenfeld, 2010). A lack of access to marriage and childrearing also has implications for how LGB people’s lives unfold, potentially creating distinct experiences in late adulthood.

LGB people’s experiences with aging may also be distinct, due to the impacts of inequality across the life course. Drawing on the insights of cumulative disadvantage theory, I emphasize that discriminatory experiences LGB people face earlier in life may be responsible for heightened challenges in late adulthood (see also Torres & Lacy, 2021). Minority stress theorists also acknowledge that stigma and discrimination lead to diminished well-being, which may be intensified among individuals who are multiply marginalized such as older, LGB people (Cyrus, 2017; McConnell et al., 2018; Meyer, 2014). Similarly, earlier homophobic experiences may compromise older LGB people’s quality of life in late adulthood.

Indeed, there is already some evidence that older LGB people face unique challenges in late adulthood. Older LGB people have smaller social networks, resulting in higher rates of social isolation, loneliness, and depression (Boggs et al., 2017; de Vries & Blando, 2004; Fredriksen-Goldsen et al., 2015; Kimmel, 2004). In addition, because many members of this community are without children and/or experienced estrangement from biological families due to homophobic discrimination, they may lack a caregiver in late adulthood (de Vries & Blando, 2004; Fredriksen-Goldsen et al., 2015). In contrast, their heterosexual counterparts regularly rely on unpaid care, especially adult children (Victor, 2005).

Simultaneously, it is possible that older LGB people are resilient and adapt well to the challenges of aging. For example, “successful aging” and “crisis competence” theories suggest that LGB people have developed strategies for combating homophobia that allow them to more effectively adjust to the aging process (Friend, 1990; Kimmel, 2004). Survey evidence suggests that nearly 75% of LGBTQ adults believe that their gender or sexual minority status has prepared them for aging (MetLife Mature Market Institute, 2010). Thus, I also ask whether LGB people adopt creative strategies to cope with aging-related challenges that arise in late adulthood.

### Visions of End-of-Life Care

Due to the possible absence of caregivers in late adulthood, older LGB people’s views on and visions of care in late adulthood are not well understood (Lottman & King, 2022; Nowakowski et al., 2019). Older LGB people often express opposition to staying in long-term care homes, due to fears about facing homophobic prejudice and discrimination from staff and other residents, in addition to anxieties about having to conceal their identity as an LGB person to avoid hostility (Brotman et al., 2003; Caceres et al., 2020; Lottman & King, 2022; Wilson et al., 2021). Some studies suggest that older LGB people will turn to partners and/or friends for care, a phenomenon that dates back to the HIV epidemic (Fredriksen-Goldson et al., 2015; Lottman & King, 2022). Many studies of this community also focus on the perspective of caregivers themselves, limiting understandings of LGB people’s views of end-of-life care (Muraco & Fredriksen, 2011). Moreover, additional research on how community commitment and involvement shape expectations for end-of-life care is needed (Muraco & Fredriksen, 2011).

Relying on data from 60 life story interviews with LGB who are 55 and older, this article investigates this unique population’s visions of care in late adulthood. I offer evidence of *queer aging*, demonstrating how *meaning-making processes* surrounding visions of aging are distinct. I also document challenges faced by this population, paying attention to how risks faced earlier in life lead to heightened aging-related challenges in late adulthood. Finally, I inspect the data for signs of resilience, including coping strategies used to combat challenges that arise.

## Research Design and Methods

I collected life story interview data during the summers of 2018 and 2019. This interviewing technique is used to gather information on a person’s life over time and pushes researchers to consider how surrounding historical contexts shape people’s lives (Atkinson, 1998). Interviews began with background questions, including nonsensitive questions about the environment they grew up in, their relationships with family early in life, and what schooling was like as a child and/or adolescent; these questions were intended to help build *rapport* and *trust* before asking more sensitive and private questions about sexuality. The second section of interviews was more open-ended, and participants were simply asked to tell their “coming out” stories. The interview ended with clarifying questions regarding the current state of one’s health, perceptions of aging, and views on end-of-life care. Although the interviews were intended to be holistic and capture information regarding a wide variety of topics (e.g., disclosure of one’s sexually minoritized status, romantic and familial relationships, discriminatory experiences), the current manuscript primarily utilizes the data that pertain to expectations regarding late life. I also probed participants when their responses were too brief. Interviews lasted roughly 1.5 hr and were audio-recorded with permission from participants.

Eligibility requirements included being 55 and older, identifying as lesbian, gay, bisexual, or queer, and residing in the Southeastern and/or Midwestern regions of the United States. The study focuses on these geographic regions because far less is historically known about older LGB people from these areas of the country. I selected participants 55 and older because these generations of LGB people were born prior to Stonewall, the beginnings of the gay-rights movements, and thus came of age prior to the growing acceptance of LGB individuals in U.S. society. The sample includes both members of the boomer and silent generation with the former coming of age during a period where LGB people experienced increased visibility, due to the Stonewall Riots. In contrast, the silent generation lived through the McCarthy era, facing fears of having their sexual identity publicly disclosed without their consent; LGB people were also often marked as criminal and as mentally ill during the time the silent generation came of age (Fredriksen-Goldsen, 2016).

Transgender and nonbinary individuals were intentionally omitted from the study, as their experiences are qualitatively different from LGB people; gender-based concerns may instead drive their current aging experiences, which would have required that a different set of interview questions be asked. Simultaneously, there may also be overlap in cisgender LGB and transgender people’s visions of late life, an issue I return to in the discussion section.

Because older LGB adults are a hard-to-reach population, I relied on non-probability sampling methods (e.g., convenience, snowball) to recruit the participants; this method is consistent with most studies of this population ( Fredriksen-Goldsen & Kim, 2017). I also relied on a feature of respondent-driven sampling methods, wherein participants are asked to have their peers contact me directly if they desired to participate in the study (see Heckathorn, 1997).

Most interviews were conducted in person or by phone in the Southeast and Midwest. Flyers containing study information were also hung at houses of worship, coffee shops, bars, etc. in a Southeastern state to recruit a diverse array of people. In addition, I recruited from social spaces from the LGB community, including online, community-based organizations, LGBTQ centers, senior centers, and retirement communities that specifically serve older LGBTQ people. I also identified gatekeepers for the study at local organizations, who helped recruit participants.

Consistent with qualitative research practices, all interviews were transcribed verbatim; participants were also assigned a pseudonym to ensure anonymity. Data were coded with ATLAS.ti, a qualitative software program. Using an interpretivist approach, I sought to discern how older LGB adults make sense of late-life plans. In doing so, my goal was to uncover meaning-making processes in the lives of participants, including motivations behind attitudes and behaviors (Krauss, 2005). In addition, I took detailed notes during and after interviews, which allowed me to enter the coding process with ideas about themes that had emerged across the interviews. I use *line-by-line* coding to identify patterns in the data that are not yet apparent and *focused coding* to collapse some of the overlapping themes into more general categories (Charmaz, 2002; Emerson et al., 1995). Finally, integrative memos were written as part of the analysis process, a practice used to record reflective thoughts about themes that are emerging in the data (Emerson et al., 1995).

Here, additional methodological reflection is in order. The use of retrospective data was crucial for documenting how risks faced earlier in life compromise quality of life in late adulthood, but it is also subject to recall bias. Nevertheless, qualitative data are inherently subjective, and the retelling of past events is rarely an objective process; stories reflect the socially constructed nature of reality (DeVault & Gross, 2012; Miller, 2019). In addition, I paid careful attention to how insider/outsider dynamics may have shaped the interview process. Although my identity as a lesbian woman helped me build rapport, I was also an outsider by virtue of my age as a woman in her early 30s. The outsider status prompted me to utilize more open-ended interviewing tactics, as it became clear that I did not know all the “right” questions to ask as a younger lesbian; using a less structured interviewing approach also boded well for gleaning deeper insight into participants’ lives. Thus, my research was an ongoing reflexive process that pushed me to capture a trustworthy and valid account of participants’ lives—one in which the view of the social actor was also honored (Cohen & Crabtree, 2008).

### Participants

At the time of data collection, participants ranged in age between 55 and 83, although the mean age is 61. Seventy-five percent of the sample are boomers, whereas 25% belong to the silent generation. Sixty-one percent of the sample identify as men. In addition, the sample is predominately white (96%). Ninety-three percent identify as gay or lesbian, while 7% are bisexual or queer. Sixty percent were married or partnered, and 35% of the sample have children. The sample is also highly educated insofar as 70% of the sample hold at least a bachelor’s degree. Moreover, the sample is largely nonreligious (61%).

## Results

### Financial Distress

Participants were asked to describe challenges facing older LGB adults, and financial distress was repeatedly named as an issue among both generations. Although poverty is common in late adulthood, it is potentially worsened by homophobic discrimination. Incidents of being denied a job and threats of being fired were faced by numerous participants. Betty (71, married, lesbian) described an incident of being denied a job at a reproductive care center, noting that HR informed her that her sexual identity was an impediment to effectively doing her job. She elaborated that HR blatantly stated that she is “going to have a really hard time finding a job in Kansas City,” due to her sexual orientation. Similarly, Fred (60, married, queer) reported that a supervisor informed him, “I’m sorry, but I [have to] let you all go … you’re too gay.” Homophobic discrimination also affected performance reviews, which has implications for promotion and salary. Dawn (73, married, lesbian) noted that homophobic bias shaped her performance review as a teacher. She revealed that her supervisor accused her of being too close to a female student, an incident she interpreted as laden with homophobic biases.

Participants’ narratives revealed that discrimination has long-term consequences on their lives, especially financial stability. Dave (80, single, gay man) noted:

Gay people have never had the advancement in jobs and all of that because of who they were … They have not built IRAs or thought about it until we were in our late 40s … we know people that just are going by the skin of their teeth. They’re on food stamps, and their job never paid enough for them to accrue anything for retirement, and their social security certainly is not good.

Similarly, Joseph (83, married, gay man) revealed that, due to a lack of promotions and bonuses, “saving was not something they [gays, lesbians, and bisexuals] could do because they could hardly live just on what they were getting. So, they enter the area of a timeframe in their lives where they don’t have the finances....” Others similarly shared that, due to a lack of stable and prosperous jobs, savings are limited. The effects of homophobic discrimination compounded, leading to further financial distress in late adulthood. Participants also later revealed that financial distress compromises their ability to pay for long-term care, a point I later revisit. Here, it becomes apparent that risks encountered earlier in life worsen well-being later in life, consistent with cumulative inequality theories (Ferraro & Shippee, 2009). These findings are also consistent with bourgeoning literature on economic stressors that emerge in late adulthood as a consequence of life-long exposure to inequality (Torres & Lacy, 2021). I return to this issue in greater depth in the discussion section.

Some variation in exposure to discrimination and subsequent financial distress was also documented among participants. Boomers were more likely than the silent generation to emphasize that discrimination in jobs and promotions over time led to financial distress, which may be due to higher rates of disclosing their identity as an LGB person. Surprisingly, married and partnered people were also twice as likely to report fears of financial distress than singles, despite the fact that they often possessed two sources of income. One possible explanation for this finding is that their partnership status rendered their sexual identity visible, making them more vulnerable to facing homophobic discrimination.

### Anxieties Surrounding Paid Care Facilities

Participants were also asked to indicate who will care for them when they are no longer able to care for themselves. Not unlike heterosexual individuals, older sexual minorities reported negative attitudes toward paid care services (Bell & Menec, 2015). However, LGB people’s aversion to long-term care settings was differentially motivated, as they feared facing homophobic abuse and discrimination by staff and residents in these facilities. Thus, how older LGB people *make sense of* end-of-life care plans potentially differs. Patricia (64, married, lesbian) asked, “… How we would be treated by the staff …? You know, would we get comments? Would we get treated differently than the other people would? [Would] other members of the community treat us poorly?” Others echoed Patricia’s concerns about the possibility of unequal treatment. Mark (69, engaged, gay man) regularly asked himself, “What if I go into a nursing home and what if they’re homophobic? Do I go back into the closet?” Here, Mark’s concerns centered on fears that he will need to conceal his identity as a gay man, not unlike how he did in earlier decades when homophobic attitudes were more rampant. Being closeted also results in a loss in feelings of autonomy, a positive view of one’s identity, and freedom of sexual expression; accordingly, one jeopardizes their right to feel mentally and physically safe at the end of life if having to conceal one’s identity (Wilson et al., 2021). Thus, some participants could not fathom the possibility of yet again concealing their identities. Moreover, it is well documented that in minority stress research that identity concealment leads to greater psychological distress (Pachankis et al., 2020). Thus, being “closeted” in long-term care homes may serve as a unique minority stressor that exacerbates depression commonly experienced among older adults and the LGB community alike.

However, attitudes toward staying in long-term care homes are also driven by fears of facing institutional and attitudinal homophobia. For example, Bill (66, separated, gay man) expressed fears that predominately heterosexual nursing homes would be comprised of individuals who “lived in an era where it wasn’t cool to be gay.” He elaborated that LGB people were viewed as mentally ill during earlier periods, which he believed would affect the attitudes of older, heterosexual residents. In addition, Kathy (68, married, lesbian) remarked that “guys with AIDS have gone through absolute hell having to live in these facilities” when asked about her thoughts on staying in a paid care setting. She elaborated that they have been physically assaulted by nurses and expressed doubts that older LGB people would receive adequate care. Thus, the historical legacy of medical mistreatment of the LGBTQ community looms large and casts doubt on medical staff’s ability to provide equitable care and treatment. In short, participants wished to avoid retraumatization in medical settings (de Vries et al., 2019).

Finally, similar to the experiences of heterosexual individuals, participants’ concerns about utilizing paid care were driven by financial instability (Cicero & Pynoos, 2016). Beth (76, partnered, lesbian) remarks that she cannot afford to stay in a long-term care home. Others discussed the availability of LGBTQ-friendly retirement communities across the country, but noted that they often are not financially accessible. Frank (68, married, gay man) remarked that even these homes “cater to the wealthy LGBT community.” He elaborated, “I don’t know why … people assume that all LGBT people have money.” As mentioned earlier, cumulative inequality largely created financial distress in late adulthood, which has spill-over effects on participants’ ability to afford housing (see also de Vries et al. 2019 for a discussion of this). Indeed, older LGB people’s exposure to poverty may be exacerbated due to the effects of discrimination on wages over time (Badgett, 2020; Grant et al., 2010).

### Aging in LGBTQ-Friendly Communities

Participants emphasized that they wished to age in community with one another or minimally with LGBTQ-friendly people in shared residences and long-term care homes; this departs from heteronormative (i.e., heterosexual-centric) understandings of aging with a predominate reliance on biological family for care. Hong (63, separated, gay man) noted that he hoped to stay in a gay-friendly long-term care home in order to avoid any potential instances of inadequate care, due to discrimination. In addition, participants also desired to live in gay neighborhoods and shared living situations, including private homes in gay neighborhoods for those who are 55+, and/or shared houses. Frank (68, married, gay man) currently resides in one of the only gay neighborhoods for 55+ people in the country. He noted that many members of his community intentionally left homophobic areas where they were “afraid to come out to people” and now embraced an opportunity to be away from individuals in their age group who are homophobic. Aging in gay-friendly communities may also ensure that participants can avoid the concealment of their identities and secure social support that is pivotal for reducing risks of social isolation, loneliness, and depression that are commonly experienced among older LGB adults (Wilson et al., 2021).

Participants envision a “cohousing revolution” in the states, where LGBTQ people provide care for one another in shared residences (see Arrigoitia Fernandez & West, 2021; Tummers & MacGregor, 2019 for more on this concept). Joseph (83, married, gay man) noted that he is part of an advocacy group for older adults, and that his group is currently “looking for facilities to put people in their own private unit where they’ll have their own private bathroom, their own bedroom, maybe a little kitchenette, and then a community dining room, a community rec room ….” Here and in other participants’ narratives emerges a discussion of an *interdependent* approach to care. Lorena (59, partnered, lesbian) and Judy (55, engaged, lesbian) both described nearly identical visions, expressing plans to build a cooperative living arrangement where women congregate, share land, garden, and provide care for each other. Similarly, Jackie (61, married, lesbian) wished her current 55+ gay-friendly neighborhood would further evolve, and stated that she would like “to be able to have somewhere where you could go to the big house. You could have a shared dinner meal … if you’re in trouble, you can call somebody and they’re there.” Moreover, Jackie also emphasized that gays and lesbians have historically “had to take care of each other...we had to stay together because no one else would.” Thus, queer history is referenced in these visions of *interdependent care* (see also de Vries et al. 2019 for a discussion of this). Moreover, participants’ visions of social support in late adulthood centered on being surrounded by other LGB people with shared culture and values.

### Aging in Place

Many participants also reported intentions to age in place and *stay in their home* as long as possible, not unlike heterosexual individuals (Bell & Menec, 2015; Cicero & Pynoos, 2016). However, their motivations for doing so were distinct, often due to fears of homophobia and heightened financial concerns. Married and partnered participants were more likely to indicate a desire to “age in place,” as it was understood that partners would help provide care. For example, Barbara (66, married, lesbian) noted that partners often provide care, but that the health and age of a partner are crucial in allowing one to age in place. However, due to a general tendency to be childfree, participants noted that friends, a partner, or a hospice care service would also help them age in their home. Roger (70, married, gay man) noted that “we have to rely on each other, whether that be spouses or family of choice.” It was well understood—even sometimes in the absence of conversation about it—that chosen family will provide care in one’s home.

Other participants shone further light on how friends provide unpaid care, which allows older LGB people to age in their own home. Barbara revealed that it is not uncommon for older gays and lesbians to be estranged from the family, which creates a situation where people must rely on friends for care. She noted, “I know many, many cases where the friends … have come together, provided food, provided transportation, been there by the bedside when somebody dies.” Teddy (63, married, bisexual man) echoed this sentiment, remarking that the absence of biological family results in older LGB people turning to friends for care.

However, the ability to age in place may not always be possible, as social isolation (e.g., lack of social networks) leaves some older LGB adults unable to live at home. Consistent with prior literature (Cicero & Pynoos, 2016), participants also acknowledged that their houses would have to be more accessible to accommodate physical decline and disability to “age in place.” Notably, this approach to care differs from “aging in LGBTQ-friendly communities” insofar as participants wish to live independently in their own homes, rather than in shared residences or long-term care homes. A similarity, however, in both approaches is an embracement of the notion of *interdependency* and a rejection of heterosexual models of care that typically include a heavy reliance on biological family and children.

### Quality Over Quantity of Life

Older LGB people also expressed a strong desire for quality over quantity of life in late adulthood. Participants, specifically, named assisted suicide as one possible method to opt for quality over quantity of life should they no longer be able to provide care for themselves. Chris (71, single, gay man) remarked, “If I got to the point where my life was not … enjoyable … if I got bedridden or something like that I’m ready to go. I don’t want to live like that. I don’t want to live in a bed. I don’t want to live on a machine … I would probably want to end my life myself.” Chris, similar to other participants, later elaborated that he is unable to accept what he perceives as a low quality of life, including an inability to complete basic bodily functions (e.g., eating, mobility). Similarly, Bill (66, separated, gay man) remarks:

… I do believe that people should have the right to end their life ... You know, I don’t consider myself suicidal, but … people should have the dignity to live their life to the fullest. And when they feel like they have no dignity, I think they should have the right to say, ‘You know, I’d like to have some medication and go to sleep.’ I mean, that’s what I would want.

The belief that one has the right to terminate their own life emerged across a range of participants.

As previously discussed, participants feared having to go back into “the closet” or facing abuse if staying in long-term care homes. Joe (60, married, queer-identified man) described LGB people’s experiences in medical facilities, noting that a number of staff “were abusive, and that a lot of the people who are residents are unaccepting.” Joe added that he is “not interested in that environment and if it gets to that point, I’d rather just kill myself.” Gloria (72, married, lesbian) also noted that she and her friends have decided that they will kill themselves before being willing to enter a nursing home. She described abusive conditions, remarking: “I’m not going to be out of it in a nursing home where you’d be drugged even more and maybe restrained and people not change you. I love life, but that’s not the life I want. That’s because they can extend life beyond what’s quality of life. And I don’t believe in that.” Here, we see that fears of ageist and homophobic abuse are prevalent among older LGB people, which push them to consider assisted suicide.

## Discussion and Implications

Using life story interview data, I offer a further articulation of “queer aging,” demonstrating that older LGB people’s visions of late life are driven by queer culture, history, and experience. Although there are some shared features of aging experiences (e.g., financial distress, opposition to staying in long-term care homes) by older adults, irrespective of sexual identity, older LGB people’s visions of late life also diverge in significant ways. Some evidence in support of cumulative inequality theories emerges, as participants reported facing heightened financial challenges in late adulthood. The *meaning* of older LGB adults’ aversion to staying in long-term care homes and subsequent preference for “aging in place” is also distinct. Participates indicate a desire to age “in place” and “in community” with the help of similar others, consistent with a tendency in queer cultures to embrace interdependent approaches to care through a reliance on chosen family. Finally, some participants discuss a desire to pursue assisted suicide, challenging mainstream values about living a long life.

This article offers some evidence of cumulative disadvantage processes among older LGB adults, filling a key gap in the literature (Torres & Lacy, 2021). Participants’ narratives suggested that the effects of job discrimination compound across the life course and heightened financial insecurity during late adulthood, making it more difficult to afford housing. Homophobic discrimination also affects retirement income and social security payments with some estimates suggesting that same-sex couples earning 34.7% less in retirement income (Badgett, 2020; Grant et al., 2010; Robinson-Wood & Weber, 2016; Rosenfeld, 2010). Due to compounding wage loss, older same-sex couples are also more likely to still be paying on a mortgage in late adulthood and are at greater risk of losing their home than their heterosexual counterparts (Rosenfeld, 2010). Moreover, older LGB people may also have to delay retirement, which could—in turn—worsen their health (Torres & Lacy, 2021). Scholars studying queer aging should continue to investigate how the accumulation of stressors over time exacerbates inequality in late adulthood (Torres & Lacy, 2021).

Simultaneously, participants’ visions of late life were laden with themes of resiliency and desires to adopt creative solutions for combating homophobic-related challenges that arise in long-term care homes; participants, specifically, mentioned that they will instead turn to other LGB people to age “in community” or “in place.” A tendency to be inventive in the face of homophobic-related challenges is not new and dates back to the HIV epidemic when LGB people embraced each other as family and a source of care in the absence of ties to biological family. However, this community-centered approach to care was not reported by participants as a deficit, but was instead viewed a means through which joy could be found in late life. Similarly, other research on older LGB adults suggests that there are unique facets of queer subculture that prepare them well for the aging process. For example, the absence of gender norms that guide spousal-care decisions among heterosexuals may actually benefit same-sex couples; indeed, gays and lesbians often coordinate medical care together as they age, and they also spend more time discussing end-of-life care plans (Reczek, 2018; Thomeer et al., 2017). Additional research on resilient and successful aging among LGB people is, thus, warranted.

The article also offers greater insight into the distinctive meanings of “aging in place” among the LGB community. Heterosexuals similarly report a desire to age and die in one’s home with dignity, which is motivated by one’s desire to maintain independence (Black et al., 2015; Thomas & Blanchard, 2009). However, older LGB people’s desires to “age in place” are driven by group-level stressors, namely fear of homophobic discrimination in long-term care homes. Thus, gerontologists should further investigate how social group membership shapes motivations behind end-of-life plans—including intentions to “age in place” (Carr, 2011). Moreover, research should further examine challenges that accompany “aging in place” among LGB people, including the possibility of worsened health in the absence of medical attention in long-term care homes (Boggs et al., 2017; Hoekstra-Pijpers, 2022). Researchers should also further consider how partnership status shapes older LGB people’s ability to “age in place,” as partnered individuals were slightly more likely to indicate a desire for this form of care. More generally, increased attention should be paid to how the late-life experiences of partnered and single LGB people vary.

*Queer aging* also entails a uniquely interdependent approach to care as reflected in participants’ reported desires to “age in community”; that is, they wish to age in close proximity to similar others via LGBT-friendly retirement communities, gay neighborhoods, and cohousing. This interdependent approach to care departs from a tendency to want to preserve independence as long as possible, which is often found among heterosexuals (Black et al., 2015; Thomas & Blanchard, 2009). Similarly, participants push for a “cohousing movement,” which would allow individuals to pool their economic and social capital and, in turn, reduce the cost of housing (Black et al., 2015; Thomas & Blanchard, 2009). Such an approach to care would benefit the aging process of older LGB adults by offsetting some of the financial distress that is the result of life-long discrimination. Moreover, this interdependent approach to care may allow older LGB people to avoid poor mental and physical health that is often a consequence of isolation in late adulthood (Thomas & Blachard, 2009). Accordingly, “aging in community” can help facilitate successful and resilient aspects of aging for older LGB adults.

Participants also reported a desire to pursue assisted suicide should they no longer be able to provide care for themselves. The findings regarding “quality over quantity of life” likely reflect the fact that aging is constructed in U.S. society as negative and a period where “doom and gloom” narratives prevail (Hareven 1995; Miller, 2019). Thus, older LGB people’s fears of aging and decline may reflect internalized ageist societal beliefs (see Kaufman & Phua, 2003; Slevin & Mowery, 2012 for more on internalized ageism among sexual minorities). Another plausible interpretation for the “quality over quantity” of life argument made by participants is that queer subculture results in LGB people departing from normative understandings of social time by rejecting the notion that they must live a long life. Scholars similarly document that some gay men engage in sexual risk-taking in exchange for sexual pleasure, a decision that is considered worthwhile even if it reduces the length of their lives (Dean, 2009). In doing so, queer theorists challenge the notion that a “good life” is one where people live forever and emphasize that some gay men would prefer to embrace a quality life. Similarly, queer individuals resist “temporal frames,” notably through breaking norms about social time, including when major life events should occur (Fabbre, 2014; Freeman, 2010; Halberstam, 2005). Thus, future studies on queer aging and the life course should carefully analyze how LGB people view time and temporality (Fabbre, 2014; Fabbre et al., 2019).

The findings also highlighted the importance of a life course perspective, especially potential generational differences (Elder, 1994). Financial distress—due to job discrimination—was more commonly reported by boomers, consistent with prior statistical studies (Fredriksen-Goldsen & Kim, 2017). The silent generation may be less likely to report discrimination, due to a tendency to conceal their identity. Indeed, this generation came of age during the McCarthy era when the President ordered federal employees to be fired (Fredriksen-Goldsen & Kim, 2017). During this same period, homosexuality was also classified by the American Psychiatric Association as a mental illness, constraining people’s ability to “come out.” In addition, boomers comprised the vast majority of participants who indicated a desire for assisted suicide to avoid negative treatment in long-term care settings; this pattern that may be explained by their observance of homophobic abuse in medical settings during the HIV epidemic. However, due to the small percentage of the silent generation in the sample, these interpretations should be approached with caution.

Similarly, cohort differences in LGB people’s experiences with late adulthood may also emerge. Marriage equality likely leads to pressures for younger LGB people to assimilate into heterosexual culture, potentially diminishing the distinctiveness of queer subcultures (Warner, 1999). Younger LGB people’s access to legal marriage, adoption, and reproductive technologies may also result in them being more likely to have biological children, who could potentially provide care to them in late adulthood. In addition, due to the liberalization of attitudes toward homosexuality, younger LGB people may have stronger ties to biological family of origin, who they could rely on as a source of care in late adulthood. Given that younger generations have grown up in a more accepting social climate, they may also be less fearful of facing discrimination in long-term care homes. Thus, the question of whether younger LGB people will face fewer aging-related challenges as it pertains to care warrants much greater attention in future research.

The study has sampling limitations, necessitating that future research further investigate how social identity characteristics shape older LGB people’s experiences. Because the study omitted transgender people, it is vital that future researchers investigate whether they experience distinctive late-life challenges. Only a small handful of studies investigate transgender people’s views of aging, with studies suggesting that they are less likely to be prepared for aging-related challenges—due to a need to use most of their time to focus on day-to-day survival in light of unemployment and poverty. Moreover, older transgender people face higher rates of loneliness and depression, financial distress, and exposure to discrimination in medical settings (de Vries et al., 2019). In addition, the current study contains few bisexual individuals, and thus future research should consider how their experiences both converge and diverge from LGB people. Finally, the sample is predominately white, and thus researchers should further examine racial variation among older LGB adults. Indeed, prior research documents that older LGB adults of color experience higher rates of discrimination in housing and employment, leading to financial distress in late adulthood; they may also have less access to social support (Kim et al., 2017; Torres & Lacy, 2021).

This article has broader implications for gerontological practice as it pertains to LGB people. It is important that staff in health care settings be educated on how to provide culturally competent care, use inclusive language, and the unique issues facing older LGBTQ patients (Lampe, 2022). This type of training is especially important in Southeastern and Midwestern regions of the United States, where it is likely that staff may harbor more homophobic attitudes and thus require greater education surrounding LGBT issues. The aforementioned cultural competency training would likely reduce homophobic discrimination in these settings, which could lead to greater utilization of these services by the LGBTQ population (Wilson et al., 2021). Greater attention to sexual diversity in end-of-life care experiences by gerontological researchers and practitioners alike would lead to enhanced quality of life for older LGBTQ people.

## References

[CIT0001] Arber, S., Davidson, K., & Ginn, J. (2003). Gender and aging: Changing roles and relationships. McGraw-Hill Education.

[CIT0002] Arrigoitia Fernandez, M., & West, K. (2021). Interdependence, commitment, learning and love. The case of the UK’s first older ­women’s co-housing community. Ageing and Society, 41(7), 1673–1696. doi:10.1017/S0144686X19001673

[CIT0003] Atkinson, R. (1998). The life story interview. Sage.

[CIT0004] Badgett, M. V. L. (2020) The economic case for LGBT equality: Why fair and equal treatment benefits us all. Beacon Press.

[CIT0005] Barbee, H. (2022). Harnessing progress: Gender, sexuality, and positive self-perceptions of aging in midlife. Journal of Aging Issues, 61, 1–9. doi:10.1016/j.jaging.2022.101008 PMC10204644 35654543

[CIT0006] Bell, S., & Menec, V. (2015). ‘You don’t want to ask for the help’ the imperative of independence: Is it related to social exclusion? Journal of Applied Gerontology, 34, NP1–NP21. doi:10.1177/0733464812469292 24652862

[CIT0007] Black, K., Dobbs, D., & Young, T. L. (2015). Aging in community: Mobilizing a new paradigm of older adults as a core social resource. Journal of Applied Gerontology, 34, 219–243. doi:10.1177/0733464812463984 25681387

[CIT0008] Boggs, J. M., Dickman Portz, J., King, D. K., Wright, L. A., Helander, K., Retrum, J. H., & Gozansky, W. S. (2017). Perspectives of LGBT older adults on aging in place: A qualitative investigation. Journal of Homosexuality, 64, 1539–1560. doi:10.1080/00918369.2016.1247539 27732524 PMC6166662

[CIT0009] Brotman, S., Ryan, B., & Cormier, R. (2003). The health and social service needs of gay and lesbian elders and their families in Canada. Gerontologist, 43, 192–202. doi:10.1093/geront/43.2.192 12677076

[CIT0010] Butler, J. (1990). Gender trouble. Routledge.

[CIT0011] Caceres, B. A., Traver, J., Primiano, J. E., Luscombe, R., & Dorsen, C. (2020). Provider and LGBT individuals’ perspectives on LGBT issues in long-term care: A systematic review. Gerontologist, 60, e169–e183. doi:10.1093/geront/gnz012 30726910 PMC7117618

[CIT0012] Calasanti, T. M., & Slevin, K. F. (2001). Gender, social inequalities, and aging. AltaMira Press.

[CIT0013] Carr, D. (2011). Racial differences in end-of-life planning: Why don’t Blacks and Latinos prepare for the inevitable? Journal of Death & Dying, 63, 1–20. doi:10.2190/OM.63.1.a 21748919

[CIT0014] Charmaz, K. (2002). Qualitative interviewing and grounded theory analysis. In J. F. Gubrium & J. A. Holstein (Eds.), Handbook of interview research (pp. 671–675). Sage.

[CIT0015] Cicero, C., & Pynoos, J. (2016). Housing’s role in the long-term care continuum. In G. D. Rowles and P. B. Teaster (Eds.), Long-term care in an aging society (pp. 231–255). Springer.

[CIT0016] Cohen, D. J., & Crabtree, B. F. (2008). Evaluative criteria for qualitative research in health care: Controversies and recommendations. Annals of Family Medicine, 6, 331–339. doi:10.1370/afm.818 18626033 PMC2478498

[CIT0017] Cyrus, K. (2017). Multiple minorities as multiply marginalized: Applying the minority stress theory to LGBTQ people of color. Journal of Gay & Lesbian Mental Health, 21, 194–202. doi:10.1080/19359705.2017.1320739

[CIT0018] Dannefer, D. (2003). Cumulative advantage/disadvantage and the life course: Cross-fertilizing age and the social science theory. Journal of Gerontology: Social Sciences, 58, S327–S337. doi:10.1093/geronb/58.6.S327 14614120

[CIT0019] de Vries, B., & Blando, J. A. (2004). The study of gay and lesbian aging: Lessons for social gerontology. In G. Herdt & B. de Vries (Eds.), Gay and lesbian aging: Research and future directions (pp. 3–28). Springer.

[CIT0020] de Vries, B., Gutman, G., Humble, A., Gahagan, J., Chamberland, L, Aubert, P., Fast, J., & Mock, S. (2019). End-of-life preparations among LGBT older Canadian adults: The missing conversations. The International Journal of Aging and Human Development, 88, 358–379. doi:10.1177/0091415019836738 30871331

[CIT0021] Dean, T. (2009). Unlimited intimacy: Reflections on the subculture of barebacking. University of Chicago Press.

[CIT0022] DeVault, M. L., & Gross, G. (2012). Feminist qualitative interviewing: Experience, talk, and knowledge. In S. N. Hesse-Biber (Ed.) The handbook of feminist research: Theory and praxis (pp. 206–236). Sage.

[CIT0023] Duggan, L. (2002). The new homonormativity: The sexual politics of neoliberalism. In R. Castronovo & D. D. Nelson (Eds.), Materializing democracy: Toward a revitalized cultural politics (pp. 175–194). Duke University Press.

[CIT0024] Edelman, L. (2004). No future: Queer theory and the death drive. Duke University Press.

[CIT0025] Elder, G. (1994). Time, human agency, and social change: Perspectives on the life course. Social Psychology Quarterly, 57, 4–15. doi:10.2307/2786971

[CIT0026] Emerson, R. M., Fretz, R. I., & Shaw, L. L. (1995). Writing ethnographic fieldnotes. University of Chicago Press.

[CIT0027] Fabbre, V. D. (2014). Gender transitions in later life: The significance of time in queer aging. Journal of Gerontological Social Work, 57, 161–175. doi:10.1080/01634372.2013.855287 24798691 PMC4051396

[CIT0028] Fabbre, V. D., Jen, S., & Fredriksen-Goldsen, K. (2019). The state of theory in LGBTQ aging: Implications for gerontological scholarship. Research on Aging, 41, 495–518. doi:10.1177/0164027518822814 30626272 PMC6760910

[CIT0029] Ferraro, K. F., & Shippee, T. P. (2009). Aging and cumulative inequality: How does inequality get under the skin? Gerontologist, 49, 333–343. doi:10.1093/geront/gnp034 19377044 PMC2721665

[CIT0030] Fingerman, K., Turiano, N. A., Davis, E., & Charles, S. T. (2013). Social and emotional development in adulthood. In J. M. Wilmoth & K. F. Ferraro (Eds.), Gerontology: Perspectives and issues (pp. 127–148). Springer.

[CIT0031] Fredriksen-Goldsen, K. I. (2016). The future of LGBT+ aging: A blueprint for action in services, polices, and research. Generations, 40(6), 16. https://www.ncbi.nlm.nih.gov/pmc/articles/PMC5375167/28366980 PMC5375167

[CIT0032] Fredriksen-Goldsen, K. I., & Kim, H.-J. (2017). The science of conducting research with LGBT older adults—An introduction to aging with pride: National Health, Aging, and Sexuality/Gender Study (NHAS). Gerontologist, 57, S1–S14. doi:10.1093/geront/gnw212 28087791 PMC5241759

[CIT0033] Fredriksen-Goldsen, K. I., Kim, H.-J., Shiu, C., Goldsen, J., & Emlet, C. A. (2015). Successful aging among LGBT older adults: Physical and mental health-related quality of life by age group. Gerontologist, 55, 154–168. doi:10.1093/geront/gnu081 25213483 PMC4542897

[CIT0034] Freeman, E. (2010). Time binds: Queer temporalities, queer histories. Duke University Press.

[CIT0035] Friend, R. A. (1990). Older lesbian and gay people: A theory of successful aging. Journal of Homosexuality, 20, 99–118. doi:10.1300/J082v20n03_07 2086655

[CIT0036] Grant, J. M., Koskovich, G., Frazer, S., & Bjerk, S. (2010). Outing age 2010: Public policy issues affecting gay, lesbian, bisexual, and transgender older adults. National Gay and Lesbian Task Force. https://www.lgbtagingcenter.org/resources/download.cfm?r=30

[CIT0037] Halberstam, J. J. (2005). In a queer time and place: Transgender bodies, subcultural lives. NYU Press.

[CIT0038] Hareven, T. (1995) Imagining the aging of men. In M. Featherstone & A. Wernick (Eds.), Images of Aging (pp. 119–134). Routledge.

[CIT0039] Heckathorn, D. D. (1997). Respondent-driven sampling: A new approach to the study of hidden populations. Social Problems, 44, 174–199. doi:10.2307/3096941

[CIT0040] Hoekstra-Pijpers, R. (2022). Experiences of older LGBT people ageing in place with care and support: A window of ordinary ageing environments, home-making practices, and meeting activities. Sexualities, 25, 25–44. doi:10.1177/1363460720936471

[CIT0041] Hull, K. E., & Ortyl, T. A. (2019). Conventional and cutting-edge: Definitions of family in LGBT communities. Sexuality Research and Social Policy, 16, 31–43. doi:10.1007/s13178-018-0324-2

[CIT0042] Kaufman, G., & Phua, V. C. (2003). Is ageism alive in date selection among men? Age requests among gay and straight men in internet personal ads. The Journal of Men’s Studies, 11, 225–235. doi:10.3149/jms.1102.225

[CIT0043] Kim, H. J., Jen, S., & Fredriksen-Goldsen, K. I. (2017). Race/ethnicity and health-related quality of life among LGBT older adults. Gerontologist, 57, S30–S39. doi:10.1093/geront/gnw172 28087793 PMC5241754

[CIT0044] Kimmel, D. C. (2004). Issues to consider in studies of midlife and older sexual minorities. In G. Herdt & B. de Vries (Eds.), Gay and lesbian aging: Research and future directions (pp. 3–28). Springer.

[CIT0045] Krauss, S. E. (2005). Research paradigms and meaning making: A primer. The Qualitative Report, 10, 758–770. doi:10.46743/2160-3715/2005.1831

[CIT0046] Lampe, N. M. (2022). Liminal lives in uncertain times: Health management during the COVID-19 Pandemic among transgender and non-binary older adults. Gerontology and Geriatric Medicine, 8, 1–8. doi:10.1177/23337214221127753 PMC9515753 36177476

[CIT0047] Lottman, R., & King, A. (2022). Who can I turn to? Social networks and the housing, care, and support preferences of older lesbian and gay people in the UK. Sexualities, 25, 9–24. doi:10.1177/1363460720944588

[CIT0048] Lytle, A., Apiceno, M. B., Dyaer, C., & Levy, S. R. (2018). Sexual orientation and gender differences in aging perceptions and concerns among older adults. Innovation in Aging, 2(3), igy036. doi:10.1093/geroni/igy036 30863795 PMC6295001

[CIT0049] McConnell, E. A., Janulis, P., Phillips, G., II, Truong, R., & Birkett, M. (2018). Multiple minority stress and LGBT community resilience among sexual minority men. Psychology of Sexual Orientation and Gender Diversity, 5(1), 1–12. doi:10.1037/sgd0000265 29546228 PMC5846479

[CIT0050] Mehrotra, C., & Wagoner, L. S. (2019). Aging and diversity: An active learning experience. Routledge.

[CIT0051] MetLife Mature Market Institute. (2010). Still out, still aging: The MetLife study of lesbian, gay, bisexual, and transgender Baby Boomers. The MetLife Market Institute. https://www.asaging.org/sites/default/files/files/mmi-still-out-still-aging.pdf

[CIT0052] Meyer, I. H. (2014). Minority stress and positive psychology: Convergences and divergences to understanding LGBT health. Psychology of Sexual Orientation and Gender Diversity, 1, 348–349. doi:10.1037/sgd0000070

[CIT0053] Miller, L. (2019). The perils and pleasures of aging: How women’s sexualities change across the life course. The Sociological Quarterly, 60, 371–396. doi:10.4324/9780429397868-81

[CIT0054] Minichiello, B., & Coulson, I. (2005). Preface: The context of promoting positive aging. In V. Minichiello & I. Coulson (Eds.), Contemporary issues in gerontology (pp. xi–xvi). Routledge.

[CIT0055] Minkler, M., & Estes, C. L. (2020). Critical perspectives on aging: The political and moral economy of growing old. Taylor and Francis.

[CIT0056] Mogle, J., & Sliwinski, M. (2013). Perspectives on cognitive aging. In J. M. Wilmoth & K. F. Ferraro (Eds.), Gerontology: Perspectives and issues (pp. 49–61). Springer.

[CIT0057] Muraco, A., & Fredriksen-Goldsen, K. I. (2011). That’s what friends do: Informal caregiving for chronically ill midlife and older lesbian, gay, and bisexual adults. Journal of Social and Personal Relationships, 28, 1073–1092. doi:10.1177/0265407511402419 24817778 PMC4013093

[CIT0058] Nowakowski, A. C. H., Chan, A. Y., Miller, J. F., & Sumerau, J. E. (2019). Illness management in older lesbian, gay, bisexual, and transgender couples: A review. Gerontological Geriatric Medicine, 5, 1–10. doi:10.1177/2333721418822865 PMC6343433 30729149

[CIT0059] Pachankis, J. E., Mahon, C. P., Jackson, S. D., Fetzner, B. K., & Branstrom, R. (2020). Sexual orientation concealment and mental health: A conceptual and meta-analytic review. Psychological Bulletin, 146, 831–871. doi:10.1037/bul0000271 32700941 PMC8011357

[CIT0060] Reczek, C. (2018) Queering aging. Syracuse University: Aging Studies Institute. https://asi.syr.edu/research/publications/queering-aging/

[CIT0061] Robinson-Wood, T., & Weber, A. (2016). Deconstructing multiple oppressions among LGBT older adults. In D. A. Harley & P. B. Teaster (Eds.), Handbook of LGBT elders (pp. 65–81). Springer.

[CIT0062] Rosenfeld, D. (2010). Lesbian, gay, bisexual, and transgender aging: Shattering myths, capturing lives. In D. Dannefer & C. Phillipson (Eds.), The Sage handbook of social gerontology (pp. 226–238). Sage.

[CIT0063] Rowe, J. W., & Kahn, R. L. (1998) Successful aging. Pantheon.

[CIT0064] Sandberg, L. J., & Marshall, B. L. (2017). Queering aging futures. Societies, 7, 1–11. doi:10.3390/soc7030021

[CIT0065] Sedgwick, E. K. (1990). Epistemology of the closet. University of California Press.

[CIT0066] Settersten, R. A., Jr., & Trauten, M. (2009). The new terrain of old age: Hallmarks, freedoms, and risks. In V. Bengtson, M. Silverstein, D. Putney, & S. Gans (Eds.), Handbook of theories of aging (pp. 455–469). Springer.

[CIT0067] Slevin, K. F., & Mowery, C. E. (2012). Exploring embodied aging and ageism among older lesbians and gay men. In L. Carpenter & J. DeLamater (Eds.), Sex for life: From virginity to viagra, How sexuality changes throughout our lives (pp. 260–277). NYU Press.

[CIT0068] Thomas, W., & Blanchard, J. (2009). Moving beyond place: Aging in community. Generations, 2, 12–17.

[CIT0069] Thomeer, M. B., Donnell, R., Reczek, C., & Umberson, D. (2017). Planning for future care and the end of life: A qualitative analysis of gay, lesbian, and heterosexual couples. Journal of Health and Social Behavior, 5, 473–487. doi:10.1177/0022146517735524 PMC5718053 29172768

[CIT0070] Torres, S., & Lacy, G. (2021) Life course transitions, personal networks, and social support for LGBTQ+ elders: Implications for physical and mental health. In A. J. LeBlanc & B. L. Perry (Eds.), Sexual and gender minority health: Advances in medical sociology (pp. 157–179). Emerald Publishing Limited Bingley.

[CIT0071] Tummers, L., & MacGregor, S. (2019). Beyond wishful thinking: A FPE perspective on communing, care, and the promise of co-housing. International Journal of the Commons, 13, 62–83. doi:10.18352/ijc.918

[CIT0072] Victor, C. (2005). The social context of ageing: A textbook of gerontology. Routledge.

[CIT0073] Warner, M. (1999). The trouble with normal: Sex, politics, and the ethics of queer life. Harvard University Press.

[CIT0074] Weston, K. (1997). Families we choose: Lesbians, gays, kinship. Columbia University Press.

[CIT0075] White, K., Bell, B. A., Huang, S. J., & Williams, D. R. (2020). Perceived discrimination trajectories and depressive symptoms among middle-aged and older Black adults. Innovation in Aging, 4(5), igaa041. doi:10.1093/geroni/igaa041 33324760 PMC7724643

[CIT0076] Wilson, K., Stinchcombe, A., & Regalado, S. M. (2021). LGBTQ+ aging research in Canada: A 30-year scoping review of the literature. Geriatrics, 6, 601–621. doi:10.3390/geriatrics6020060 PMC8293146 34204715

